# Supplementary material for: tRNA m1A modification regulate HSC maintenance and self-renewal via mTORC1 signaling
Source: Nat Commun. 2024 Jul 8;15:5706. doi: 10.1038/s41467-024-50110-9 (PMC11231335; doi:10.1038/s41467-024-50110-9)
Supplement: Supplementary file 1 — Supplementary Information [file 41467_2024_50110_MOESM1_ESM.pdf]

Supplementary Figures

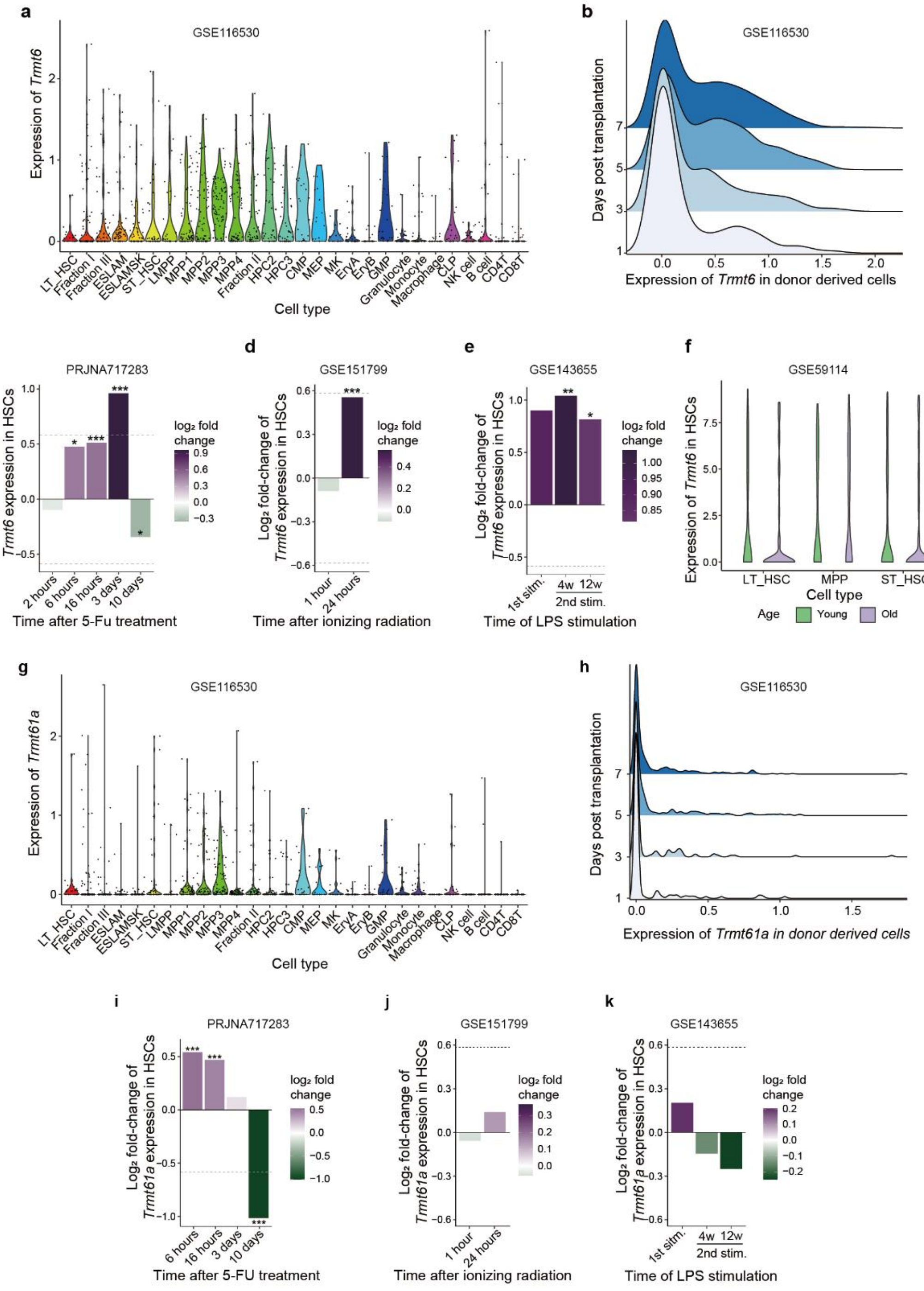

Figure S1. *Trmt6/Trmt61a* were dysregulated in HSC upon different treatment or during aging.

a. Violin plot showing expression of *Trmt6* in mouse hematopoietic cells (GSE116530).

b. Ridgeline plot showing expression change of *Trmt6* in mouse HSCs after transplantation (GSE116530).
c. Bar graph showing expression changes of *Trmt6* in mouse HSCs several hours or days after 5-FU treatment (PRJNA717283).
d. Bar graph showing expression changes of *Trmt6* in mouse HSCs several hours after ionizing radiation (GSE151799).
e. Bar graph showing expression changes of *Trmt6* in mouse HSCs after LPS stimulation (GSE143655).
f. Violin plot comparing expression of *Trmt6* between HSCs of young and old mice (GSE59114).
g. Violin plot showing expression of *Trmt61a* in mouse hematopoietic cells (GSE116530).
h. Ridgeline plot showing expression change of *Trmt61a* in mouse HSCs after transplantation (GSE116530).
i. Bar graph showing expression changes of *Trmt61a* in mouse HSCs several hours or days after 5-FU treatment (PRJNA717283).
j. Bar graph showing expression changes of *Trmt61a* in mouse HSCs several hours after ionizing radiation (GSE151799).
k. Bar graph showing expression changes of *Trmt61a* in mouse HSCs after LPS stimulation (GSE143655).

In c-e and i-k, ***, adjusted p value < 0.001; **, adjusted p value < 0.01; *, adjusted p value < 0.05.

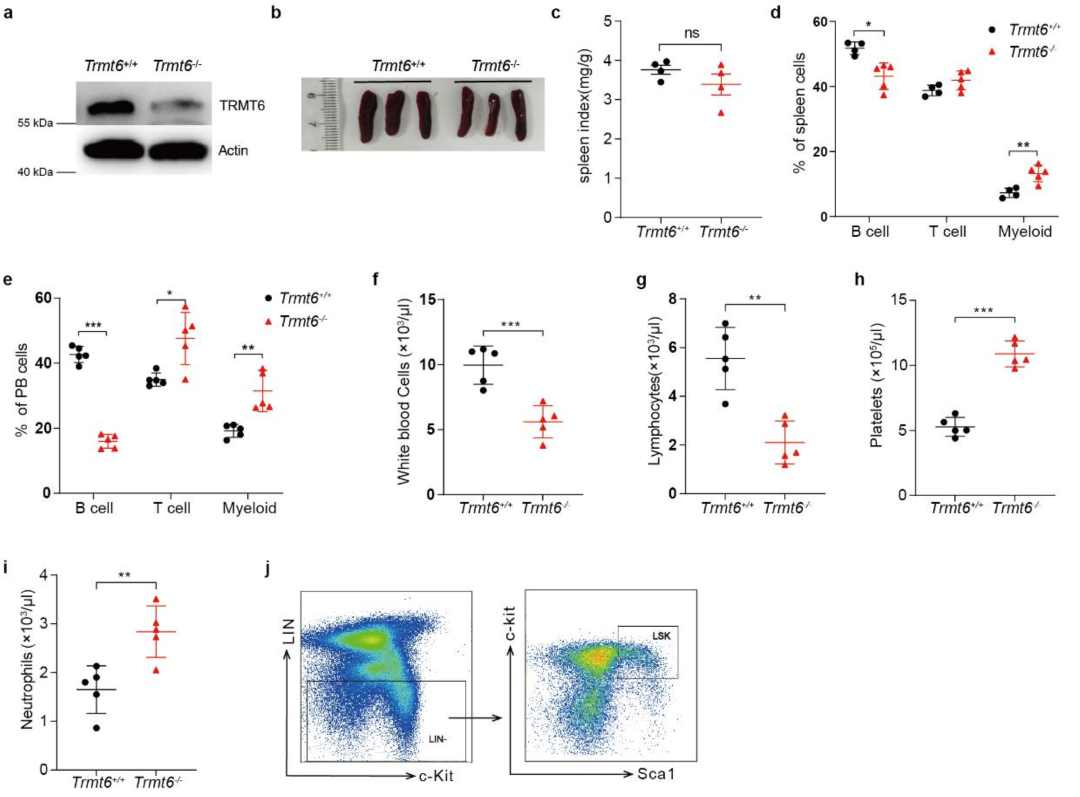

Figure S2. Characterization of *Mx1-Cre Trmt6* conditional KO mice.

a. Western blotting analysis of TRMT6 expression in LSK ($Lin^{-}c\text{-}Kit^{+}Sca\text{-}1^{+}$) cells from *Trmt6*$^{-/-}$ and *Trmt6*$^{+/+}$ mice; β-Actin was used as a loading control.

b. Representative photograph of spleens from *Trmt6*$^{-/-}$ and *Trmt6*$^{+/+}$ mice. n=5 mice per genotype.

c. Spleen index from *Trmt6*$^{-/-}$ and *Trmt6*$^{+/+}$ mice. n=5 mice per genotype.

d. Frequency of myeloid cells ($CD11b^{+}$), B cells, T cells in the spleens of *Trmt6*$^{-/-}$ and *Trmt6*$^{+/+}$ mice. n=5 mice per genotype.

e. Frequency of myeloid cells ($CD11b^{+}$), B cells, T cells in PB of *Trmt6*$^{-/-}$ and *Trmt6*$^{+/+}$ mice. n=5 mice per genotype.

f-i. Complete blood count (CBC) parameters 1 week after polyI:C injection. n=5 mice per genotype.

j. Representative FACS graphs for sorting LSK cells (HSPCs).

Data represent the mean ± SD from three independent experiments. *, $P<0.05$; **, $P<0.01$; ***, $P<0.001$. For all the above statistics, p-values were obtained using unpaired parametric two-tailed t-test. Exact P values are provided as Source Data.

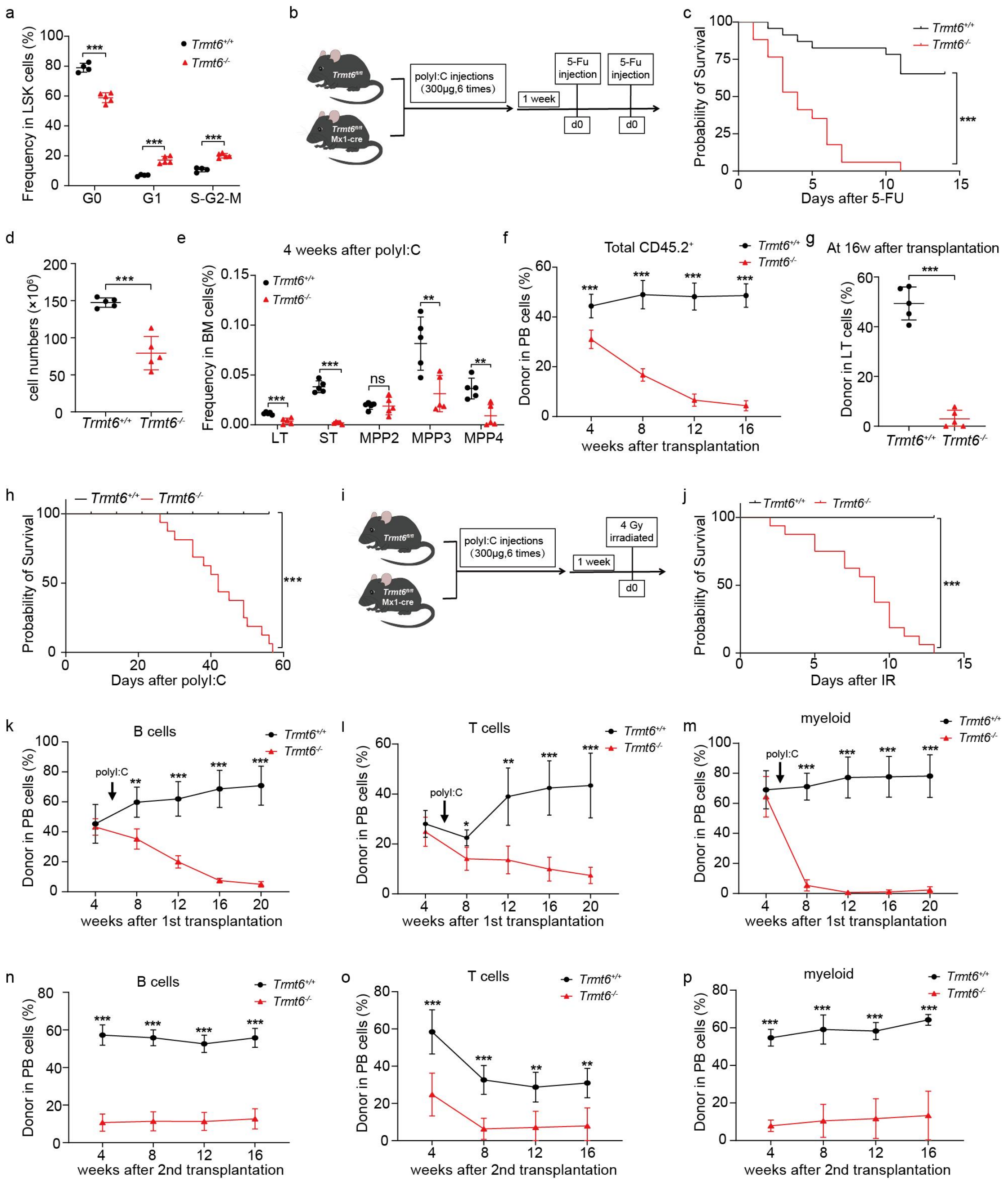

Figure S3 *Trmt6* is necessary to maintain the self-renewal and regenerative ability of hematopoietic stem cells

a. Frequency of the cell cycle distribution in *Trmt6*$^{-/-}$ and *Trmt6*$^{+/+}$ LSKs are shown. n=5 mice per genotype.

b-c. Experimental scheme and survival curve of *Trmt6*$^{-/-}$ (n=17 mice) and *Trmt6*$^{+/+}$ (n=23 mice) mice following sequential 5-FU treatment.

d. Whole bone-marrow cellularity of *Trmt6*$^{-/-}$ and *Trmt6*$^{+/+}$ mice (n=5 mice per genotype).

e. Proportion of LT, ST, MPP2, MPP3, MPP4 in the bone marrow of mice 4 weeks after polyI:C injection.

f. Sorted *Trmt6*$^{+/+}$ and *Trmt6*$^{-/-}$ mice (CD45.2^{+}) LT-HSC were mixed with CD45.1^{+} bone marrow cells and transplanted into recipient mice (CD45.1^{+}), and the percentage of CD45.2^{+} cells in the peripheral blood was examined at 4-16 weeks.

g. Proportion of donor-derived (CD45.2^{+}) cells in LT-HSC at 16 weeks post-transplantation

h. Survival curve of *Mx1-Cre*;*Trmt6*$^{fl/fl}$ (*Trmt6*$^{-/-}$,) mice (n=18 mice) and control mice(n=19 mice) after polyI:C injection.

i-j. Experimental scheme and survival curve of *Trmt6*$^{-/-}$ and *Trmt6*$^{+/+}$ mice following 4Gy irradiated (n=16 mice per group).

k-m. Percentage of donor-derived B cells, T cells and myeloid cells at the indicated time points in the first competitive transplantation. *Trmt6*$^{+/+}$, n=4 mice and *Trmt6*$^{-/-}$ *mice,* n=5 mice.

n-p. Percentage of donor-derived B cells, T cells and myeloid cells at the indicated time points in the secondary competitive transplantation, n=5 mice per genotype.

Data represent the mean ± SD from three independent experiments. *, P<0.05; **, P<0.01; ***, P<0.001. For all the above statistics, P values were obtained using unpaired parametric two-tailed t-test. Exact P values are provided as Source Data.

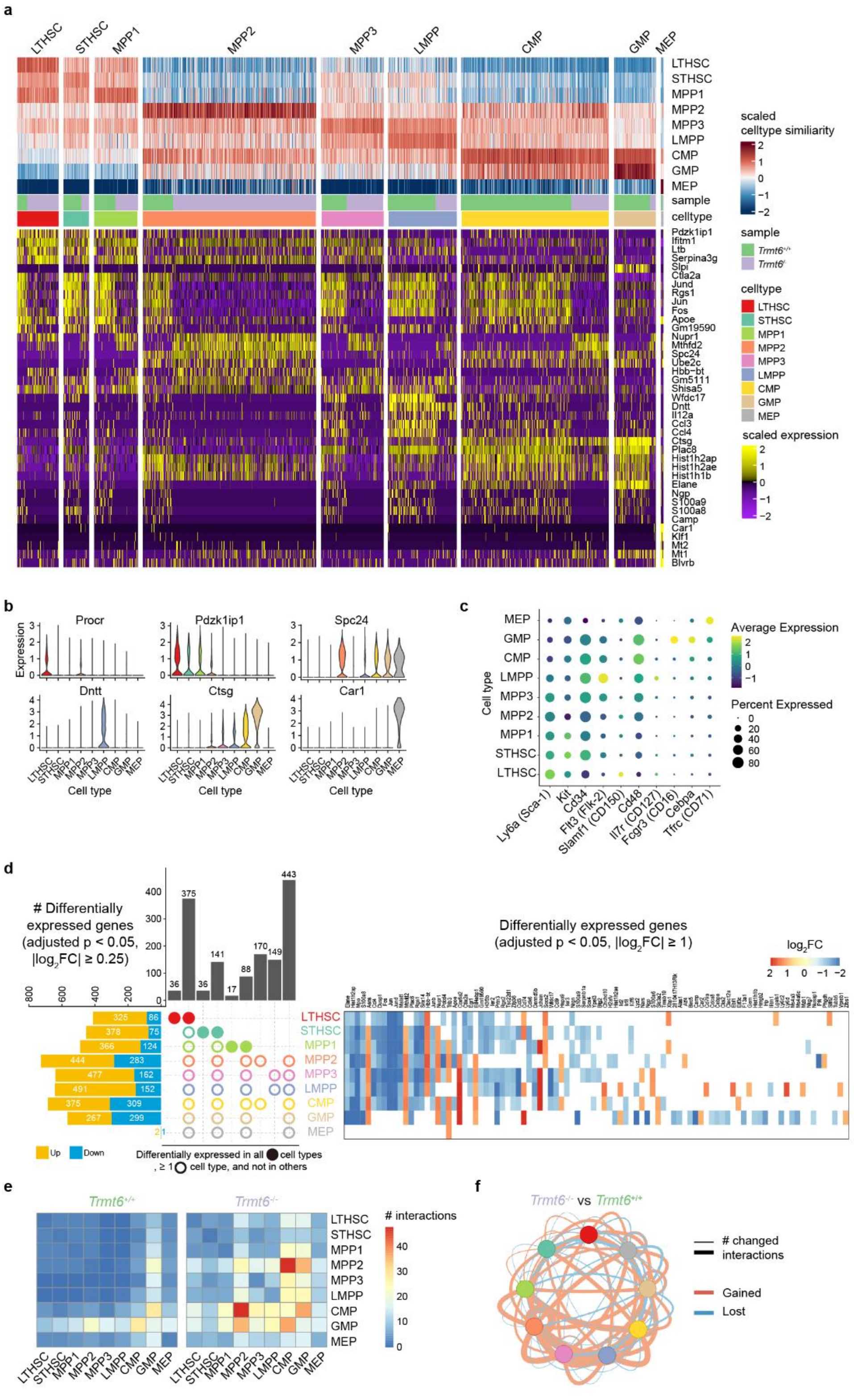

Figure S4. scRNA-seq reveals dysregulation of genes and over-activation of cell-cell communication upon *Trmt6* knockout.

a. Heat map of top signature gene expression for each cell populations annotated with Nestorowa *et al.* data. Scaled cell type similarity scores to Nestorowa *et al.* data are annotated on top.

b. Violin plot showing the expression of selected genes which specifically highly expressed in one or few cell populations.

c. Dot plot showing the expression of known markers of HSPCs.

d. Differentially expressed genes in each cell population between *Trmt6*$^{-/-}$ and *Trmt6*$^{+/+}$ mice. Left: Bar graphs showing the number of up- and down-regulated genes upon *Trmt6* depletion in each cell population identified with a more relax threshold and the size of overlap among them. Right: heatmap differentially expressed genes upon *Trmt6* depletion in each cell population identified with a more stringent threshold.

e. Heatmaps showing the number of interaction pairs between indicated bone marrow cell populations in *Trmt6*$^{-/-}$ and *Trmt6*$^{+/+}$ mice. The color key indicates the interaction counts.

f. A network showing the changes in cell-cell interactions across all cell populations in *Trmt6*$^{-/-}$ and *Trmt6*$^{+/+}$ mice. The width of the lines indicates the changed interaction counts, and gained and lost interaction are presented in orange and blue, respectively.

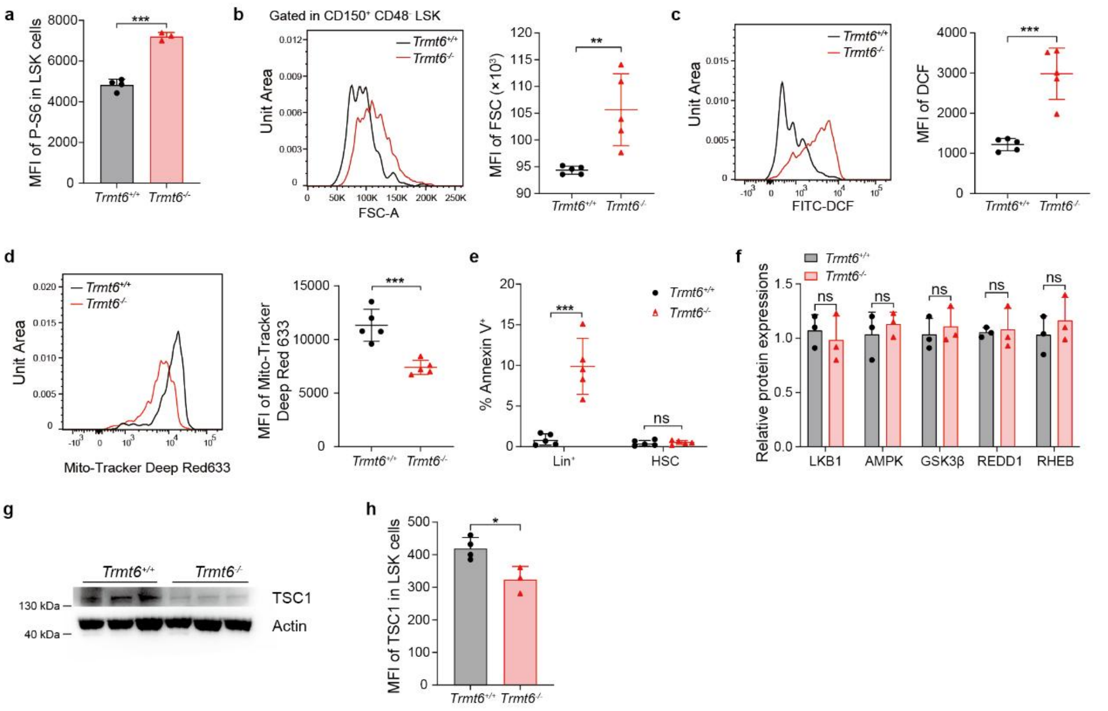

Figure S5. *Trmt6*$^{-/-}$ HSC shows the molecular features of mTOR activation and ROS production.

a. Median fluorescence intensity analysis of p-S6 levels in the LSKs of *Trmt6*$^{-/-}$ and *Trmt6*$^{+/+}$ mice. n=5 mice per genotype.

b. Flow analysis of FSC changes in HSC (CD150$^+$CD48$^-$ LSK) peak plot (left) and statistical plot (right). n=5 mice per genotype.

c. Flow analysis of HSC (CD150$^+$CD48$^-$ LSK) with peak ROS changes (left) and statistical plots (right). n=5 mice per genotype.

d. Flow analysis of CD150$^+$CD48$^-$ LSK with peak mitochondrial membrane potential changes (left) and statistical plots (right). n=5 mice per genotype.

e. Proportion of Annexin V$^+$ cells of *Trmt6*$^{-/-}$ and *Trmt6*$^{+/+}$ mice one week after polyI:C injection. n=5 mice per genotype.

f. The relative protein levels of LKB1, AMPK, GSK3β, REDD1and RHEB in *Trmt6*$^{-/-}$ LT-HSCs vs. *Trmt6*$^{+/+}$ LT-HSCs are shown. n=5 mice per genotype.

g. TSC1 protein expression in *Trmt6*$^{+/+}$ and *Trmt6*$^{-/-}$ HSPCs.

h. Median fluorescence intensity analysis of TSC1 levels in the LSKs of *Trmt6*$^{-/-}$(n=3 mice) and *Trmt6*$^{+/+}$(n=4 mice) mice.

Data represent the mean ± SD from three independent experiments. ns, *P* value ≥ 0.05; *, *P*<0.05; **, *P*<0.01; ***, *P*<0.001. For all the above statistics, *P* values were obtained using unpaired parametric two-tailed t-test. Exact *P* values are provided as Source Data.

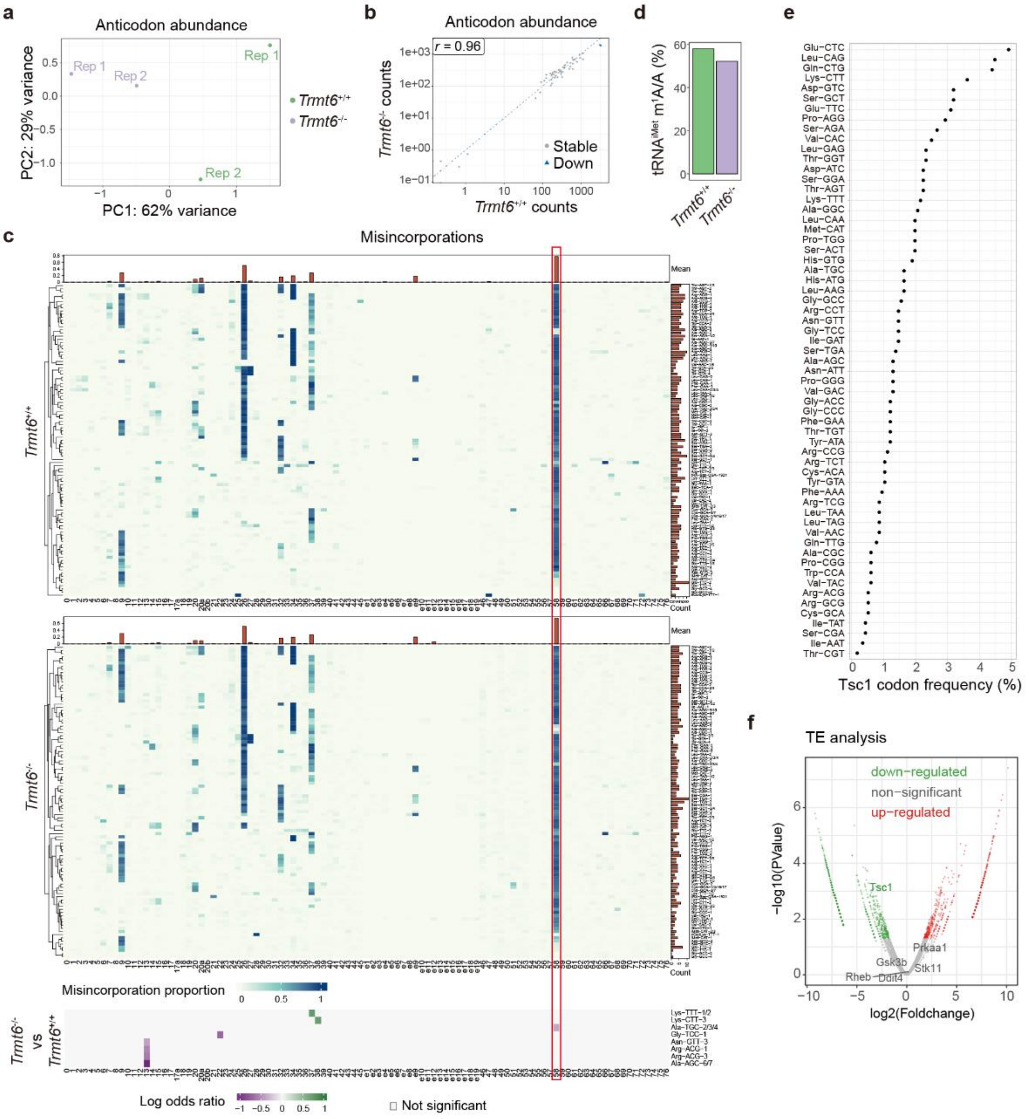

Figure S6 TRMT6-mediated tRNA modification promotes *Tsc1* translation.

a. PCA plot of tRNA expression detected by mim-tRNA-seq in *Trmt6*$^{+/+}$ and *Trmt6*$^{-/-}$ HSPCs.

b. Scatter plot of tRNA expression detected by mim-tRNA-seq in *Trmt6*$^{+/+}$ and *Trmt6*$^{-/-}$ HSPCs. Grey and blue indicate stable and down-regulated expression in *Trmt6*$^{-/-}$ HSPCs, respectively. *r*, Pearson correlation efficient.

c. Heatmap of modification level per tRNA transcript in *Trmt6*$^{+/+}$ (top) and *Trmt6*$^{-/-}$ (middle) HSPCs and their changes (bottom) detected by mim-tRNA-seq. (n = 2;

top bar graph, mean misincorporation per position; right bar graph, number of sites per transcript with detectable misincorporation signatures in ≥ 10% of reads spanning that position).

d. mim-tRNA-seq detection of the m^1A58 modification level of $tRNA^{iMet}$ in *Trmt6*$^{+/+}$ and *Trmt6*$^{-/-}$ HSPCs. n = 2 biologically independent samples.

e. The codon frequency of mouse *Tsc1* mRNA.

f. TE (FPKM in Ribo-Seq divided by FPKM in input RNA sequencing) analysis of mRNA translation between *Trmt6*$^{+/+}$ and *Trmt6*$^{-/-}$ HSPCs.

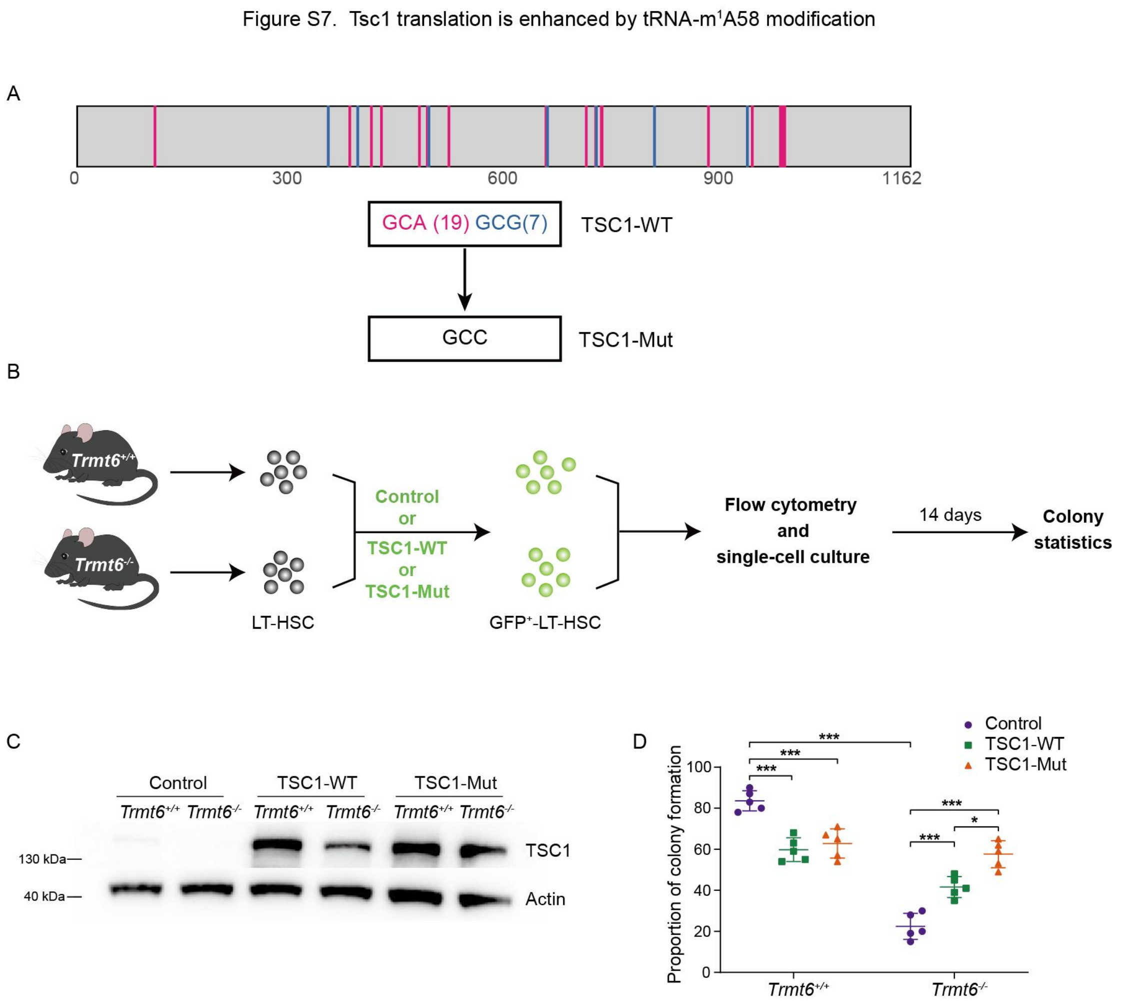

Figure S7. *Tsc1* translation is enhanced by tRNA-m^{1}A58 modification

a. Schematic diagram of the *Tsc1* codon-switch assay.

b. Lentivirus-mediated expression of TSC1-WT and TSC1-mutant (TSC1-Mut) in *Trmt6*$^{-/-}$ and *Trmt6*$^{+/+}$ HSCs.

c. Protein levels of TSC1 were quantified by immunoblotting. Representative data of two independent experiments are shown.

d. LT-HSCs were sorted from (B) and assessed by the CFU assays. Colonies were counted 12 days later. n=5 mice per genotype. Data represent the mean ± SD from three independent experiments. ns, *P* value ≥ 0.05; *, *P*<0.05; **, *P*<0.01;

***, $p<0.001$. The *P* values were obtained using one-way ANOVA followed Dunnett's multiple comparisons test. Exact *P* values are provided as Source Data.

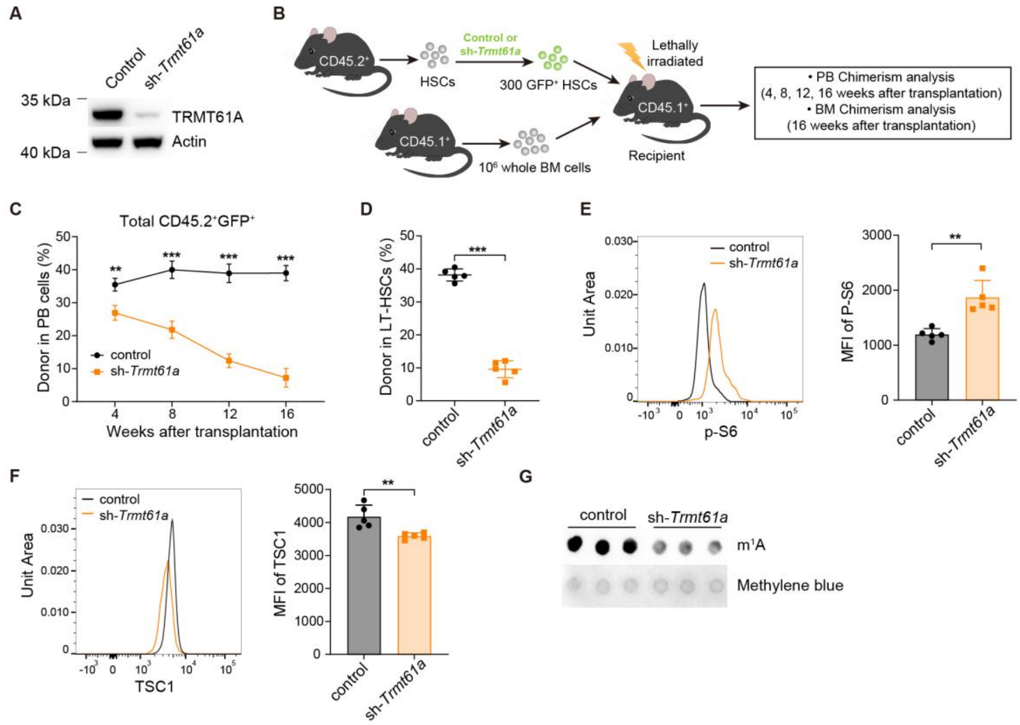

Figure S8. Characterization of *Trmt61a*-knockdown HSCs.

a. Western blot assay for sh-*Trmt61a* knockdown efficiency.

b. Experimental scheme for competitive transplantation with sh-*Trmt61a* or control lentivirus infected HSCs.

c. The percentage of GFP$^+$ (sh-*Trmt61a* or control lentivirus infected HSCs) cells in the peripheral blood was examined at 4-16 weeks. n=5 mice per genotype.

d. The percentage of GFP$^+$ cells in LT-HSC was detected 16 weeks after transplantation. n=5 mice per genotype.

e. Flow cytometric analysis of p-S6 changes in GFP$^+$ cells in HSCs after infected sh-*Trmt61a* or control lentivirus 3 days, peak plots (left) as well as statistical plots (right). n=5 mice per genotype.

f. Flow cytometric analysis of changes in TSC1 protein levels in GFP^+ cells in HSCs 3 days after infected sh-*Trmt61a* or control lentivirus, peak plots (left) as well as statistical plots (right). n=5 mice per genotype.

g. Dot-blot analysis of changes in tRNA m^1A modification levels in GFP^+ cells in LSK 4 days after infected sh-*Trmt61a* or control lentiviral.

Data represent the mean ± SD from three independent experiments. *, $P<0.05$; **, $P<0.01$; ***, $P<0.001$. For all the above statistics, *P* values were obtained using unpaired parametric two-tailed t-test. Exact *P* values are provided as Source Data.

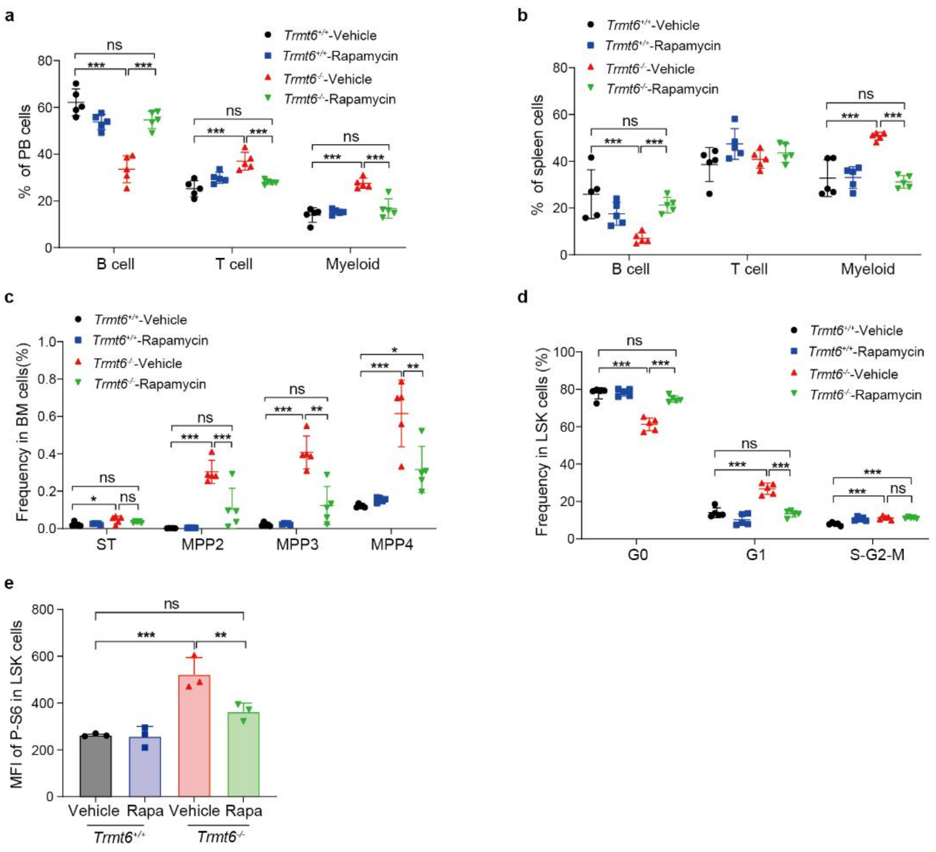

Figure S9 Rapamycin alleviates activation of mTORC1 and partially rescue the function of *Trmt6*-deficient HSCs

a. Frequency of B cells, T cells, myeloid cells ($CD11b^{+}$) in PB of *Trmt6*$^{-/-}$ and *Trmt6*$^{+/+}$ mice after mTOR inhibitor rapamycin treatment *in vivo.* n=5 mice per genotype.

b. Frequency of B cells, T cells, myeloid cells ($CD11b^{+}$) in spleen of *Trmt6*$^{-/-}$ and *Trmt6*$^{+/+}$ mice after mTOR inhibitor rapamycin treatment *in vivo.* n=5 mice per genotype.

c. Frequency of ST-HSCs, MPP2, MPP3 and MPP4 in BM cells are shown after mTOR inhibitor rapamycin treatment *in vivo.* n=5 mice per genotype.

d. Frequency of the cell cycle distribution in *Trmt6*$^{-/-}$ and *Trmt6*$^{+/+}$ LSKs are shown after mTOR inhibitor rapamycin treatment *in vivo.* n=5 mice per genotype.

e. Median fluorescence intensity analysis of p-S6 levels in the LSKs of *Trmt6*$^{-/-}$ and *Trmt6*$^{+/+}$ mice after mTOR inhibitor rapamycin treatment *in vivo*. n=5 mice per genotype.

Data represent the mean ± SD from three independent experiments. ns, *P* value ≥ 0.05; *, *P*<0.05; **, *P*<0.01; ***, *P*<0.001. For all the above statistics, *P* values were obtained using one-way ANOVA followed Dunnett's multiple comparisons test. Exact *P* values are provided as Source Data.

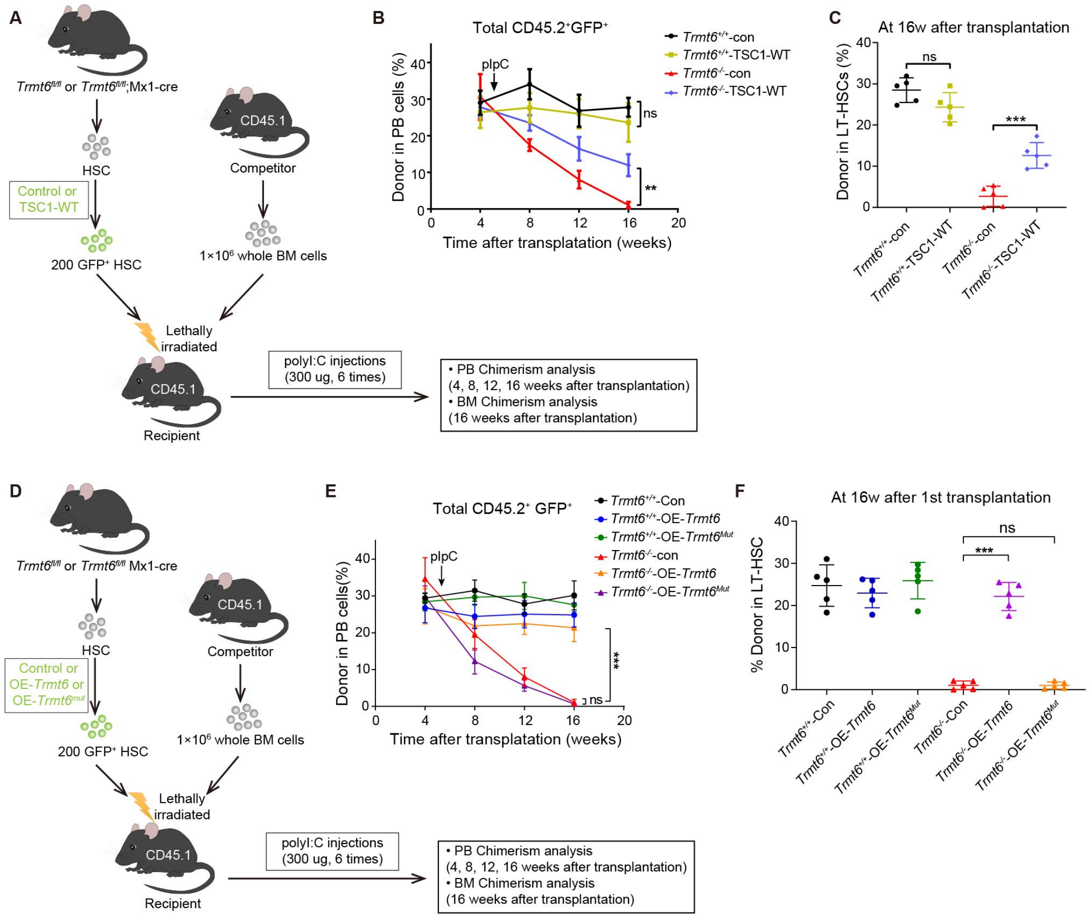

Figure S10. Overexpression (OE) of TSC1 and TRMT6 but not enzyme-dead TRMT6 partially rescued the function of *Trmt6*-deficient HSCs.

a. Experimental scheme for competitive transplantation with OE-TSC1 in *Trmt6*$^{-/-}$ and *Trmt6*$^{+/+}$ HSCs.

b. Proportion of donor-derived (CD45.2^{+}) GFP^{+} cells in peripheral blood 4-16 weeks after transplantation.n=5 mice per genotype.

c. Proportion of donor-derived (CD45.2^{+}) GFP^{+} cells in LT-HSC at 16 weeks post-transplantation.n=5 mice per genotype.

d. Experimental scheme for competitive transplantation with OE-*Trmt6* or OE-*Trmt6*^{Mut} in *Trmt6*$^{-/-}$ and *Trmt6*$^{+/+}$ HSCs.

e. Proportion of donor-derived ($CD45.2^+$) GFP^+ cells in peripheral blood 4-16 weeks after transplantation.n=5 mice per genotype.

f. Proportion of donor-derived ($CD45.2^+$) GFP^+ cells in LT-HSC at 16 weeks post-transplantation.n=5 mice per genotype.

Data represent the mean ± SD from three independent experiments. ns, *P* value ≥ 0.05; *, *P*<0.05; **, *P*<0.01; ***, *P*<0.001. For all the above statistics, *P* values were obtained using one-way ANOVA followed Dunnett's multiple comparisons test. Exact *P* values are provided as Source Data.
